# 2120 Long-acting reversible contraceptive uptake in female sex workers and single mothers in Rwanda and Zambia

**DOI:** 10.1017/cts.2018.292

**Published:** 2018-11-21

**Authors:** Jessica Li, Rachel Parker, Kristin Wall, Lisa Haddad, Susan Allen

**Affiliations:** Emory University

## Abstract

OBJECTIVES/SPECIFIC AIMS: Long-acting reversible contraception (LARC) has been well established as the most cost-effective form of contraception, but LARC usage in developing countries remains low. As part of a multi-center parent study on HIV incidence, we implemented an integrated family planning program to increase LARC uptake in single women in Rwanda and Zambia. We aim to evaluate rates of LARC uptake, LARC discontinuation and incident pregnancy following family planning counseling. METHODS/STUDY POPULATION: We enrolled 3 cohorts of single sexually active HIV-negative women between the ages of 18–45 years: single mothers (SM) in Zambia, female sex workers (FSW) in Zambia and FSW in Rwanda. Participants were followed every 3 months for up to 5 years. At each visit, we discussed fertility goals and counseled participants on HIV risk reduction and contraceptive options. Eligible participants (not pregnant, already using a LARC method, or using a permanent contraceptive method) were offered a LARC method, specifically the copper IUD or Jadelle implant. Data was collected on demographic factors, sexual behavior, sexual and reproductive history, and gynecological exams and laboratory tests were performed if necessary. RESULTS/ANTICIPATED RESULTS: In total, 458 Rwandan FSW, 555 Zambian FSW, and 521 Zambian SM were enrolled, with a median follow-up time of 6 months, 12 months, and 9 months, respectively. Accounting for any LARC uptake during longitudinal follow-up, our preliminary results show an increase in LARC usage from 21% at screening to 51% at the end of follow-up among Rwandan FSW, an increase from 12% to 42% in Zambian FSW and an increase from 18% to 44% in Zambian SM. We hypothesize that demographic factors (e.g., younger age, higher education level) and sexual history (e.g., greater number of sexual partners, any STIs or reproductive health disturbances) will be associated with increased rates of LARC uptake. We also hypothesize that LARC users will have significantly lower proportions of contraceptive method discontinuation and incident pregnancy compared to non-LARC users. DISCUSSION/SIGNIFICANCE OF IMPACT: FSW and SM are disproportionately affected by high rates of unintended pregnancy, which can lead to obstetric complications and poor psychosocial outcomes. It is imperative that family planning interventions in developing countries target these populations to overcome obstacles in reproductive health and promote gender equality. Our study will provide necessary insights to an integrated family planning program, which will guide future efforts to design, implement and evaluate family planning initiatives for high-risk populations.

